# The Rescue and Rehabilitation of Koalas (*Phascolarctos cinereus*) in Southeast Queensland

**DOI:** 10.3390/ani6090056

**Published:** 2016-09-15

**Authors:** Emily Burton, Andrew Tribe

**Affiliations:** 1 School of Agriculture and Food Sciences, University of Queensland, Gatton QLD 4343, Australia; emily.burton@uqconnect.edu.au; 2 The Gainsdale Group, P.O. Box 108, Fortitude Valley, Brisbane QLD 4006, Australia

**Keywords:** koalas, rehabilitation, release

## Abstract

**Simple Summary:**

Little is understood about the overall success of current wildlife rehabilitation techniques and the implications of these as an effective conservation strategy. This study collated admission records from four major wildlife hospitals catering to sick and injured koalas across southeast Queensland from 2009 to 2014, and analyzed specific factors that may be important when quantifying the extent and effectiveness of this work. The study found koalas to be at an increased risk from urbanization and human disturbance, that various rehabilitation techniques are employed amongst the four wildlife hospitals, and that a majority of koalas are either euthanized or die whilst in care rather than being released back to the wild. These results provide an interesting insight into current koala rehabilitation practices and have important implications for further research to better understand the practice of rescue and rehabilitation as an effective conservation strategy for this species.

**Abstract:**

Koala populations in southeast Queensland are under threat from many factors, particularly habitat loss, dog attack, vehicle trauma and disease. Animals not killed from these impacts are often rescued and taken into care for rehabilitation, and eventual release back to the wild if deemed to be healthy. This study investigated current rescue, rehabilitation and release data for koalas admitted to the four major wildlife hospitals in southeast Queensland (Australia Zoo Wildlife Hospital (AZWH), Currumbin Wildlife Sanctuary Hospital (CWH), Moggill Koala Hospital (MKH) and the Royal Society for the Prevention Against Cruelty to Animals Wildlife Hospital at Wacol (RSPCA)), and suggests aspects of the practice that may be changed to improve its contribution to the preservation of the species. It concluded that: (a) the main threats to koalas across southeast Queensland were related to urbanization (vehicle collisions, domestic animal attacks and the disease chlamydiosis); (b) case outcomes varied amongst hospitals, including time spent in care, euthanasia and release rates; and (c) the majority (66.5%) of rescued koalas were either euthanized or died in care with only 27% released back to the wild. The results from this study have important implications for further research into koala rescue and rehabilitation to gain a better understanding of its effectiveness as a conservation strategy.

## 1. Introduction

The koala (*Phascolarctos cinereus*) is one of Australia’s most recognizable and high profile native species. However, koala populations are under serious threat as a result of rapid land clearing and associated urbanization pressures, particularly habitat loss, dog attack, vehicle trauma and disease. 

Loss of suitable koala habitat remains the most significant threat to the continued survival of the species across all parts of its original range [1], leaving the animal particularly susceptible due to its specialist folivore diet of eucalyptus leaves [2]. In addition, habitat destruction often forces koalas to disperse, and so become vulnerable to vehicle strikes and domestic animal attacks [3]. 

This situation is particularly apparent in southeast Queensland, where massive urban development and habitat fragmentation have severely harmed native wildlife populations [4]. It is estimated that there are currently less than 35,000 koalas in the southeast Queensland region [4], with population declines of up to 51% in less than three years [5]. 

These threats to koalas have been reflected in the numbers coming into care at wildlife hospitals in southeast Queensland. For instance, approximately 300 sick, injured and orphaned koalas are rescued, rehabilitated and released back to the wild each year from the Koala Coast region [5], an area recognized as important koala habitat, and includes portions of Brisbane, Logan City and the Redland City Council areas [6]. However, few studies have monitored the rescue and rehabilitation of koalas in Queensland and none have examined the trends in koala admission and outcomes.

Wildlife rescue and rehabilitation is common in many countries and can be an effective tool for treating sick, injured and orphaned animals. The ultimate goal of wildlife rehabilitation is the “successful” transition and return of an individual back to its natural habitat, in a fit and healthy state to reproduce and perform all other natural functions and behaviours that benefit the environment and the species [7]. However, the effectiveness of wildlife rehabilitation as a conservation strategy remains unclear and important aspects of the process (e.g., post-release survival rates) are not easily assessed [7]. 

This study aimed to collate koala admission records from the four major wildlife hospitals in southeast Queensland that specialize in treating koalas and to analyze specific factors which may be important in quantifying koala rehabilitation activities for scientific research. These factors include patient demographics, the cause of admission, the time spent in care, and the outcomes of rescue and rehabilitation. 

## 2. Materials and Methods 

### 2.1. Data Collection

Four wildlife hospitals in southeast Queensland specialise in treating sick and injured koalas; Australia Zoo Wildlife Hospital (AZWH), Currumbin Wildlife Sanctuary Hospital (CWH), Moggill Koala Hospital (MKH) and the Royal Society for the Prevention Against Cruelty to Animals Wildlife Hospital at Wacol (RSPCA). 

Hospital admission databases from these four wildlife hospitals were accessed for the period from January 2009 to June 2014. 

### 2.2. Data Organization and Management 

The following information regarding each koala was recorded (a) hospital; (b) identification number “ID”; (c) sex; (d) age group; (e) cause of admission; (f) date of admission; (g) time in care; (h) outcome. 

### 2.3. Limitations of the Data

Data collection indicated that some information was inaccessible or unavailable. If possible the missing records were collected from an alternative source [8] and if no other source was available, information from previous years were used to extrapolate admission data to obtain a complete database for the study period. This was done to allow for a more informed investigation of the current trends in koala admissions.

### 2.4. Data Analysis 

The wildlife hospital admission records were examined using simple descriptive analyses including graphs and tables compiled using Microsoft Excel 2010. This was deemed the simplest way of displaying and examining the data, as other statistical analyses were considered inappropriate and unnecessary for this study, which did not test specific hypotheses. 

## 3. Results

Several abbreviations are used in this section to simplify reading, including the names of the four wildlife hospitals: Australia Zoo Wildlife Hospital (AZWH), Moggill Koala Hospital (MKH), Currumbin Wildlife Hospital (CWSH) and the Royal Society for the Prevention against Cruelty to Animals (RSPCA) Wildlife Hospital. In addition, “Temp” is used for “temperature degrees Celsius”, admissions caused by trauma vehicle strike are “VS” and domestic animal attacks are “DAA” respectively. 

### 3.1. Number of Koalas Admitted

**Figure 1:**
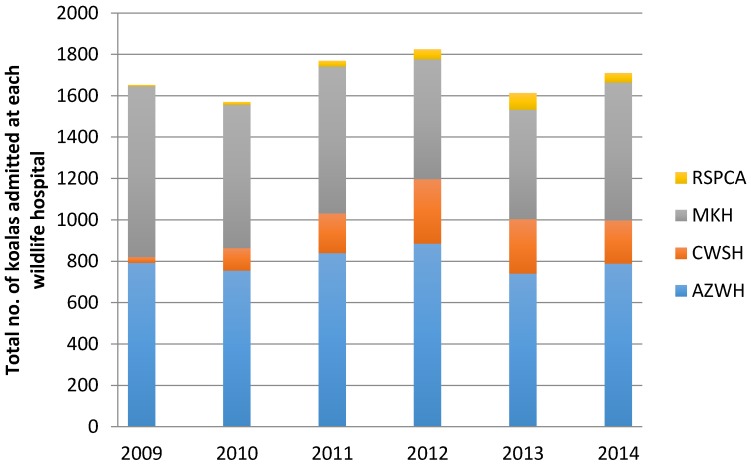
The number of koalas admitted to each wildlife hospital for the years 2009 to 2014.

Figure 1 shows the number of koalas admitted to the four wildlife hospitals for each year from 2009 to 2014. 

This results indicate that during the six-year study period 10,139 koalas were admitted to the four wildlife hospitals in southeast Queensland. More than 1600 koalas were rescued annually with a peak of 1825 in 2012. Approximately 85% were admitted to AZWH and MKH. The results also show that the number being admitted each year remained stable over this six year period. More than 80% of koalas admitted were adult (81.5%) and the proportion of males to females was almost equal.

**Figure 2:**
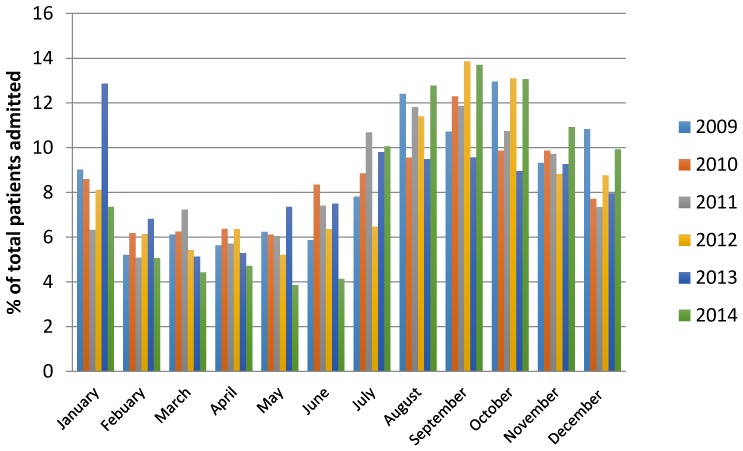
Seasonal trends of koalas admitted to the four wildlife hospitals throughout the study period.

Figure 2 displays seasonal trends of koalas admitted to the four wildlife hospitals between 2009 to 2014, including annual proportions as well as monthly contributions. 

The results indicate that more koalas are brought into care during the spring to early summer, and that conversely the number reduces from February to July.

### 3.2. Causes of Koala Admissions

**Figure 3:**
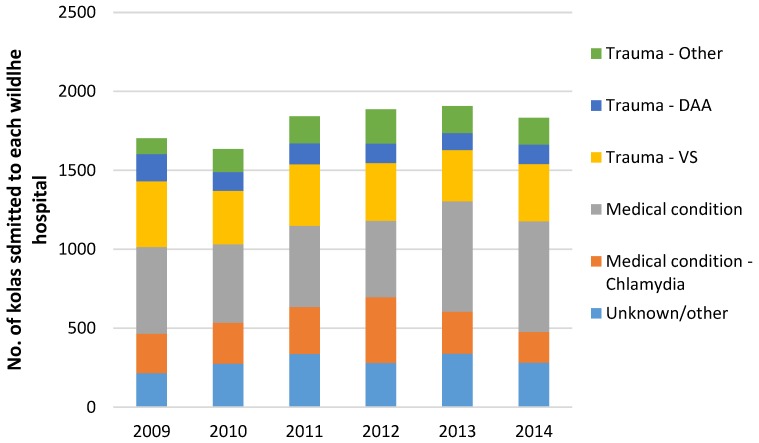
The causes of admission of koalas to each wildlife hospital for the years 2009 to 2014.

The causes of admission of koalas across the four wildlife hospitals during the study period are shown in Figure 3. 

These results indicate that more than half of the koalas were admitted for a “medical condition”, of which more than 196 had been diagnosed as “chlamydiosis” (22%). A further 38% had suffered from “trauma”, of which more than half were caused by vehicle strikes and about 20% from attacks by domestic animals. However, in many cases the data do not indicate a definitive diagnosis or identification of precise admission causes was not available.

### 3.3. Time Spent in Care

**Figure 4:**
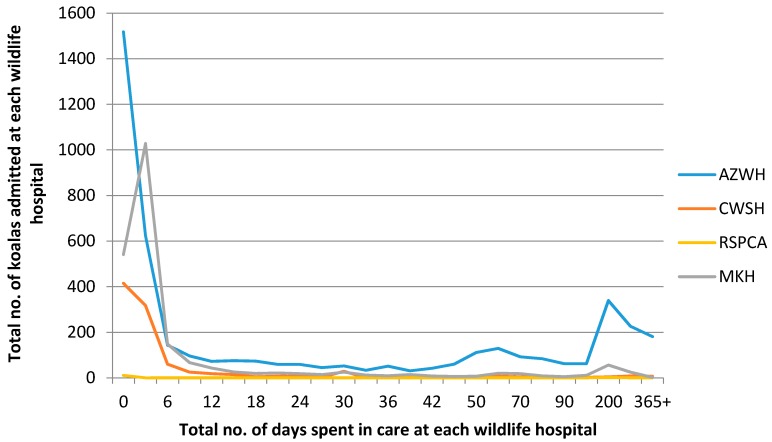
The time koalas spent in care at each wildlife hospital over the six years of the study period.

The time the koalas spent in care at each wildlife hospital (see Figure 4) indicates that most were in core care (the basic, first level of treatment including diagnosis a patient receives upon admittance) less than one week. The increase seen between days 150 and 365+ is largely an artefact of the change in the *x*-axis scale, but does indicate that some koalas did spend an extended amount of time in care, particularly at AZWH.

### 3.4. Outcomes for the Admitted Koalas

**Figure 5:**
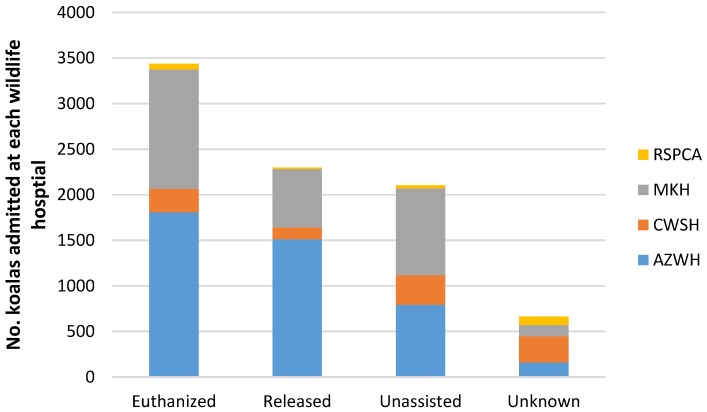
The outcomes for all koalas admitted to the four wildlife hospitals for the period 2009 to 2014 (euthanized, released, unassisted deaths whilst in care and unknown or unrecorded outcomes).

There were essentially two outcomes for koalas admitted to the wildlife hospitals: those animals assessed as being able to survive in the wild were released, and those considered unsuitable for release which were euthanized or that died whilst in care. The four sub-classifications of outcomes from each patient across the four hospitals is presented in Figure 5, and includes those that were euthanized, individuals successfully released back to the wild, those that encountered an unassisted death whilst in care and outcomes that were unknown or unrecorded. 

Almost two thirds of koalas (65.5%) which came into care were either euthanized or died, while 27% were able to be released. In total, 3437 koalas were euthanized by the four wildlife hospitals, representing 40% of the total admitted. Comparatively, 2297 (27%) koalas were released back into the wild throughout the study period.

## 4. Discussion

### 4.1. Admission Rates of Koalas into Wildlife Hospitals

Approximately 10,000 koalas were admitted across the four wildlife hospitals throughout the six-year study period, with approximately 1600 admitted per annum. The largest intake of koalas was seen during 2012, where over 1800 animals were admitted (see Figure 1). 

The AZWH admitted the most koalas in the study period with 4804 animals. This was followed by the MKH with 4004 koalas, CWSH with 1107, and the RSPCA 224. It was expected that AZWH and the MKH would have the largest intake of koalas due to their specialist care of the species and the facilities available for treating and holding koalas over longer periods. 

Intake variation may also be attributed to other factors including operation hours, which vary between establishments (AZWH and RSPCA are open 24/7 whilst the MKH and CWSH have differing opening hours throughout the week). Admissions to AZWH, CWSH and the RSPCA all remained relatively constant during the study period, while the MKH koala admissions slightly decreased over time (see Figure 1). This decline may be attributed to a number of factors including a decline in koalas within the areas of southeast Queensland that are most frequently served by MKH, or that more koalas were taken to the other hospitals for unknown reasons. 

The results also show that the number of koalas being admitted each year remained stable over the six-years. However, evidence suggests that the overall koala population in southeast Queensland has decreased significantly. In 2013 Wildlife Queensland conducted a survey of koala numbers throughout the Koala Coast region of southeast Queensland and found that it had decreased considerably in recent years. The population was estimated to be 6246 throughout the region during 1999, but was found to have dropped to 2279 by 2008, a 64% decline over ten years [9]. 

Therefore, it can be inferred that whilst koala populations seem to be in decline across the region, koala admission rates remain steady or increasing due to factors including continued urbanization and the threats associated with the growing human population within the area. Ultimately, we can presume these pressures to be having a relative impact upon koala populations and their ability to cope with rising challenges including domestic dog attacks and vehicle collisions. At the same time, it may be that people are becoming increasingly aware of koalas living in their local communities and actively rescuing sick, injured and orphaned individuals. Of course if the total population of free-living koalas in southeast Queensland continues to decline, this will eventually reduce the numbers being admitted to wildlife hospitals.

The admissions data also demonstrate a seasonal effect with more koalas being admitted during the spring and early summer months (Figure 2). This is consistent with the koala breeding season, when more individuals are dispersing to find mates, and in the process are more prone to becoming victims of vehicle or domestic animal attack.

### 4.2. Causes of Admission 

Rural and urban koala populations in southeast Queensland have decreased, fundamentally influenced by human-related activities including domestic dog attacks and vehicle collisions, along with other naturally occurring factors including bushfires [10]. This finding was somewhat supported by the evidence from this study. Direct and indirect human impacts were a primary influence on admissions rates through vehicle collisions and domestic animal attacks, however the results from this study also found medical conditions, particularly chlamydiosis to be the major cause of admission (see Figure 3). 

The results indicate that more than half of the koalas were admitted for a “medical condition”, of which 196 had been diagnosed as “chlamydiosis” (12%). However, 40% of the other “medical condition” admissions comprised undiagnosed conditions, and many of these may have involved chlamydiosis but cannot be confirmed due to the lack of detailed medical records for these cases.

This is supported by the prevalence of the disease from other research projects conducted throughout southeast Queensland, with infection rates of chlamydiosis in koalas ranging from 43 to 57% within wild populations in southeast Queensland [11]. 

A further 38% had suffered from “trauma”, of which more than half were caused by vehicle strikes and about 20% from attacks by domestic animals. 

Most causes of admission, including vehicle strikes appeared to remain relatively consistent throughout the study period, with some instances including domestic animal attacks even decreasing over time. However, chlamydiosis appeared to increase in prevalence from 2009 to 2012 followed by a steady decline from 2013 to 2014 (see Figure 3). 

It is reasonable to suggest that disease associated with rapid urbanization in southeast Queensland is increasing affecting wild koala populations, and this may be of concern for the future viability of the species in this region. This is supported by the finding that 20% of all koala admissions to the Port Macquarie Wildlife Hospital in New South Wales were due to clinical signs of chlamydiosis (conjunctivitis and cystitis), second to admissions caused by trauma [12]. According to Dique et al. [13], unless there is proper planning to mitigate further habitat fragmentation, it is likely that the local population of koalas will continue to be threatened by chlamydiosis (which is believed to be exacerbated by stress), vehicle strikes and domestic dog attacks.

However, it is also apparent that in many cases the data did not allow for a definitive diagnosis or identification of the precise cause of admission. These inconsistencies in the dataset demonstrate poor record keeping, likely to reduce the accuracy of research into koala rehabilitation in wildlife hospitals. 

The development of better admittance record keeping systems (particularly databases) and protocol is one of the most influential areas that would facilitate both higher quality and broader scope research to develop better management practices. An example format could be a universal database accessible from an online webpage (stored using cloud technology). The use of drop-down menus is highly recommended (rather than typed entry) to facilitate analysis and report functions although the option to add notes, especially if a value of other or unknown were selected would also be useful. Context sensitive language and features would help increase usability. It is also recommended that multiple entry fields be utilized for cause of arrival/history to better account for causation.

### 4.3. Time Spent in Care 

Figure 4 shows that most koalas were in core care less than one week across the four wildlife hospitals. This is consistent with the average length of stay of wildlife patients across Victorian shelters, with less than 5 days for 6% of admissions, followed by 29% after 10 days, 21% after 25 days and only 7% were still in care after 100 days [14]. 

However, AZWH also showed a significantly prolonged time in care for some animals, with 17% of koala patients still in care after two hundred days, and some for longer than a year. This finding is consistent with a recent news article claiming AZWH spent two years to successfully rehabilitate a single koala that had contracted chlamydiosis [14]. AZWH maintained the prolonged treatment of the animal provided invaluable research opportunities into chlamydiosis, and how to improve treatment regimens for future patients [15]. 

However, other researchers suggest prolonged rehabilitation can jeopardise survival following release therefore the process should be as short as possible, and some animals should be euthanized on first examination rather than being allowed to die in captivity at a later time [14].

### 4.4. Outcomes of Rehabilitation

Most (66.5%) rescued koalas were either euthanized or died in care with only 27% returned to the wild. 

Similar studies of wildlife rehabilitation in both Australia and the United States show that more than 40% of animals could be released to the wild [14] compared with the 27% found in this study for koalas. Studies conducted on similar marsupial species describe a range of both optimistic and pessimistic outcomes, some results found no difference in release methods of hand-reared eastern grey kangaroos and the overall outcome of rehabilitation efforts a relative success [16], whilst others found that almost all hand-reared ringtail possums to be taken by predators soon after release [17]. 

As the wild koala population in southeast Queensland continues to decline, the fact that only one in four koalas admitted across the major wildlife hospitals are successfully rehabilitated and suitable for release is likely to be of increasing concern in the future. Similarly, due to the current lack of objective information regarding post-release survival rates, it remains unclear as to what effect current rescue and rehabilitation techniques are having upon southeast Queensland koala populations.

The relatively small proportion of successfully released koalas throughout the study period may be simply due to the severity of admission causes or the difficulty in treating and successfully rehabilitating koalas. Nonetheless, it does highlight the need for further research and resources to improve current treatment and rehabilitation regimens.

The results also show that the rates of release and euthanasia found amongst the four wildlife hospitals varied: AZWH released 35% and euthanized 42%, MKH released 21% and euthanized 43%, CWSH released 13% of its admitted koalas and euthanized 26% and the RSPCA released 7% of admitted koalas and euthanized 31% (see Figure 5). Such variations may suggest that there are differences in the treatment regimens employed by each hospital. This finding is consistent with similar research that found case outcomes for koalas admitted with chlamydiosis differed across wildlife hospitals [12]. 

Outcome decisions at koala hospitals are largely based on clinical signs rather than including diagnostic tests [12]. This can be problematic because underlying health issues can go unidentified or untreated, also this may encourage only the treatment of clinical signs and ignore core, complex diagnosis and subsequently void all treatment efforts. Case outcomes based on clinical signs alone are likely to cause problems for the health of individual animals and the population, reducing fertility and long term survival of koala populations [12]. By ignoring subclinical signs of chlamydiosis, koala hospitals are likely overlooking valuable opportunities to decrease infection rates and the relative effects of disease in local koala populations [12].

Similarly, findings from investigated case outcomes at three major wildlife hospitals in the southeast Queensland and northern New South Wales concluded that different standards and procedures were commonly employed [18]. In addition, the three facilities also seemed to base their treatments almost entirely on the presence of clinical signs of disease, rather than on diagnostic testing to determine other underlying factors that may be affecting the animal [18]. 

Other evidence suggests wildlife hospitals differ in how they manage and treat koalas, highlighted by a recent disagreement between koala rescue groups in southeast Queensland. Moreton Bay Koala Rescue (MBKR) admits all their rescued koalas to the AZWH [19], but Pine Rivers Koala Care (PRKC) prefers the government-run MKH. Rescuers from the MBKR believe that the AZWH has a higher survival rate but rescuers from PRKC dispute this with comparable survival rates from the MKH [19]. 

Other evidence to suggest differences in attitudes and protocol in regards to case outcomes is highlighted in a news article by Fraser [19] that suggests AZWH on average spends up to $5000 on medical treatment for each koala admitted into their facilities. Similarly, one koala recently admitted into the AZWH spent two years in care due to ongoing effects of chlamydiosis, with treatment costs of the animal reaching $20,000 [19].

Consequently, some researchers conclude that rehabilitation and treatment procedures for koalas need to be specifically addressed to minimize wastage, maintain high animal welfare standards and ensure its effectiveness for both the rehabilitated individual and the local resident population to which they will return [12]. They maintain that despite their financial and regulatory restraints, wildlife hospitals should be reevaluating their rehabilitation and treatment protocols to develop consistent and effective treatment regimens of each koala and population. 

## 5. Conclusions 

This study has provided some interesting and relevant information about the current rescue and rehabilitation techniques of sick and injured koalas across southeast Queensland, which will subsequently contribute to a better understanding of how these methods benefit as an effective conservation strategy. 

Firstly, admission rates of koalas to the major wildlife hospitals appear to be steady over time, whilst the major causes of admission were largely influenced by human intervention (including vehicle collisions and domestic animal attacks), as well as the disease chlamydiosis. However, the lack of accuracy and specificity in the admission data is a weakness which is likely to reduce the effectiveness of research on koala rehabilitation in wildlife hospitals. The results show that the mortality rates of koalas in hospital care far exceeds survival rates. 

The results also suggest that there may be differences in the admission, treatment and rehabilitation protocols adopted by the wildlife hospitals in southeast Queensland which are contributing to the differences in the euthanasia and release rates for koalas from each hospital. 

However, with the current lack of objective information about post release survival rates, it is unclear what effect the current rescue and rehabilitation techniques are actually having on southeast Queensland koala populations. Recommendations from this study include:

- A uniform database program or structure with a dedicated database manager at each wildlife hospital to effectively record standard, accurate admission information in order to facilitate investigations and analyses.
- Further research into the most effective rehabilitation options should be undertaken to evaluate outcomes and success rates for the long-term survival of koalas.
- Research should also be conducted to investigate stress levels on patients admitted for lengthy periods of time (longer than seven days), and if this is likely to have an effect on the welfare of those animals whilst in care and on their survival when released back into the wild.
- More post-release monitoring of released koalas to assess not only their survival back in the wild but also their contribution to the sustainability of the remaining wild populations.
